# Influence of Alumina Grade on Sintering Properties and Possible Application in Binder Jetting Additive Technology

**DOI:** 10.3390/ma16103853

**Published:** 2023-05-19

**Authors:** Maciej Kwiatkowski, Joanna Marczyk, Piotr Putyra, Michał Kwiatkowski, Szymon Przybyła, Marek Hebda

**Affiliations:** 1 Faculty of Materials Engineering and Physics, Cracow University of Technology, Warszawska 24, 31-155 Kraków, Poland; maciej.kwiatkowski@createc.com.pl (M.K.); joanna.marczyk@pk.edu.pl (J.M.); s.przybyla@createc.com.pl (S.P.); 2 Createc Sp. z o.o., E. Kwiatkowskiego 9, 37-450 Stalowa Wola, Poland; m.kwiatkowski@createc.com.pl; 3 Research Network Lukasiewicz-Krakow Institute of Technology, ul. Zakopiańska 73, 30-418 Kraków, Poland; piotr.putyra@kit.lukasiewicz.gov.pl

**Keywords:** Al_2_O_3_, particle size, sinterability, specific surface area, SPS, 3D printing

## Abstract

Alumina is one of the most popular ceramic materials widely used in both tooling and construction applications due to its low production cost, and high properties. However, the final properties of the product depend not only on the purity of the powder, but also, e.g., on its particle size, specific surface area, and the production technology used. These parameters are particularly important in the case of choosing additive techniques for the production of details. Therefore, the article presents the results of comparing five grades of Al_2_O_3_ ceramic powder. Their specific surface area (via Brunauer–Emmett–Teller (BET) and Barrett–Joyner–Halenda (BJH) methods), particle size distribution, and phase composition by X-ray diffraction (XRD) were determined. Moreover, the surface morphology was characterized by the scanning electron microscopy (SEM) technique. The discrepancy between generally available data and the results obtained from measurements has been indicated. Moreover, the method of spark plasma sintering (SPS), equipped with the registration system of the position of the pressing punch during the process, was used to determine the sinterability curves of each of the tested grades of Al_2_O_3_ powder. Based on the obtained results, a significant influence of the specific surface area, particle size, and the width of their distribution at the beginning of the Al_2_O_3_ powder sintering process was confirmed. Furthermore, the possibility of using the analyzed variants of powders for binder jetting technology was assessed. The dependence of the particle size of the powder used on the quality of the printed parts was demonstrated. The procedure presented in this paper, which involves analyzing the properties of alumina varieties, was used to optimize the Al_2_O_3_ powder material for binder jetting printing. The selection of the best powder in terms of technological properties and good sinterability makes it possible to reduce the number of 3D printing processes, which makes it more economical and less time-consuming.

## 1. Introduction

Alumina (Al_2_O_3_) is one of the most important engineering materials from the group of oxide ceramics widely used in industry [1]. Depending on the intended application, it is used both in its pure form and in combination with other materials [2]. The α-Al_2_O_3_ from the crystallographic point of view consists of oxygen ions that form a close-packed, almost hexagonal structure with aluminum ions filling the octahedral gaps [3,4]. Aluminum oxide attracts the attention of researchers due to its unique properties and high functional parameters, e.g., high hardness and exceptional wear resistance. In addition, it has remarkable mechanical properties at elevated temperatures and good chemical and thermal stability [5,6]. It is an important material not only because of its excellent properties but also because of its biocompatibility. Therefore, alumina ceramics have been widely used in the aviation, aerospace, automotive, medicine, and military industries [3,7]. Al_2_O_3_ can be used as a tool and as a construction material, which is characterized by a high melting point, compressive strength, corrosion resistance, and a high modulus of elasticity [8,9]. Alumina is also sometimes used by researchers as a material reinforcing the properties of metal matrices, such as aluminum [10,11], copper [12,13], magnesium [14], or titanium [15]. However, a certain limitation in the applicability of this material is its brittleness [10]. It is well known that it is possible to sinter technical ceramics in the form of alumina. Different techniques are used for Al_2_O_3_ production which allows for the control of the desired properties of the final product through the changes in grain size, density, or porosity [1]. The most commonly used methods for consolidating Al_2_O_3_ powders are cold pressing with conventional sintering and hot pressing. However, Orlova [16] showed that traditional methods were characterized by the limited efficiency of ceramics compaction. For this reason, the idea of looking for alternative ways of ceramics production that will allow for the formation of a homogeneous structure of high density becomes very important. Particular attention should be paid to high-speed sintering methods by passing high-power current pulses, for example, spark plasma sintering (SPS) [16]. The SPS technique is widely used due to the rapid densification not only in the production of ceramics but also for such materials as metals or composites [17,18]. Spark plasma sintering combines a uniaxial mechanical load and constant or pulsed electrical current to consolidate powder materials [19,20]. The SPS technique is based on the rapid heating rates and short residence times of the powders in an inert ambient or in a vacuum by applying a pulsed direct current to the sintered powder and the mold. The sintering temperature is monitored by a thermocouple placed on the outer surface of the mold and via a pyrometer [3,16]. The main advantages of the SPS method compared to conventional sintering are the reduced sintering time and the ability to control the parameters of the sintering process and the material microstructure [21,22]. The use of short residence times and high heating rates prevent the excessive growth of crystallites. Therefore, due to the possibility of compaction with limited grain growth, SPS has the potential to obtain dense and fine microstructures with better mechanical properties [1,3,23,24]. Wang et al. [25] showed that SPS is an effective method for producing fine-grained, high-density Al_2_O_3_ ceramics from a powder with a smaller particle size. This is facilitated by the use of a high heating rate. However, this does not apply to large powder particles of about 20 µm. In this case, its driving force for densification is too low. For example, researchers such as Rivero-Antúnez et al. [26] have used the SPS technique to obtain alumina matrix composites reinforced with graphene oxide, which has significant potential to improve the strength, electrical conductivity, or thermal stability of the product [27,28]. Most scientific publications describe the optimization and testing of various sintering processes for powder materials, but, generally, they were carried out for expensive materials. The best results in terms of sintering efficiency were noticeable for Taimei’s TM-DAR powder. However, its mass use for commercial applications is associated with high costs, which is unfavorable for economic reasons. Therefore, cheaper alternatives are still sought [29]. Another important issue is the geometry of the manufactured products. Ceramics with complex shapes are difficult to produce by conventional methods due to the necessity to use molds, which increases the time and cost of production [30,31,32]. Additionally, the machining of ceramic elements is problematic due to their high brittleness and hardness. Defects such as cracks can not only arise in ceramic parts. Cutting tools are also subject to severe wear [31]. These problems can be eliminated by using additive manufacturing (AM) technology to fabricate ceramic parts. AM is defined as the process of joining materials using the layer-by-layer method according to the data from the three-dimensional (3D) model [33]. The 3D printing of ceramics was first described by Marcus and Sachs in the 1990s [31]. Currently, ceramic materials are processed by multiple additive manufacturing techniques. For ceramics production, researchers use methods such as stereolithography (SLA) [34], fused deposition modeling (FDM) [35], direct ink writing (DIW) [36], selective laser sintering (SLS) [37], and binder jetting (BJ) [38,39]. Compared to other techniques, binder jetting is considered a suitable method to produce ceramic parts due to its ability to build complex geometries [25].

Binder jetting is the process of creating three-dimensional objects using powdered materials, which are based on inkjet technology. The process consists in spreading the powder layer by layer and selectively depositing binder droplets to create an object. The next step is burning off the binder, and then subjecting the green parts to a sintering or infiltration process in order to obtain the desired mechanical properties. The BJ method provides ease of scale-up and is characterized by a high building rate and compatibility with a wide range of materials compared to other AM technologies [40,41].

In the case of ceramic powders used for the binder jetting process, their mechanical properties, such as bulk density and flowability, are particularly important. These properties will be influenced by the geometrical properties of the powder, such as particle shape and size distribution. Currently, researchers for printing use ceramic powders of irregular and spherical shapes. The flowability of ceramic powders affects their printability. Even the distribution of the powder layer will result in a complete structure. In the case of the lower flowability of the powder, it will be less evenly distributed, which may lead to reduced properties. The shape of ceramic powders determines their flowability, packing density, and pore structure. Spherical particles are characterized by a higher bulk density and better flowability compared to irregular particles [40]. Oropeza et al. [42] showed that the use of irregularly shaped powders in AM resulted in lower powder bed densities than for spherical powders. This is due to both poorer particle packing as well as increased intermolecular friction. Typically, such powder beds not only have a low packing density but also show cracks and defects. As a result, for angularly shaped and weakly flowable powders, the bed roughness is usually higher [41,43]. This is confirmed by the results obtained by Chen et al. [43]. The shape of the ceramic particles has a significant influence on sinterability [44]. Powders with large particle sizes, compared to finer powders, show better flowability, but worse sinterability. The finer particle size (especially <20 μm), due to their high surface area and higher energy state, leads to higher densities and a faster sintering process [40,41,43]. In contrast, studies by Suwanprateeb et al. [29] showed that the printed parts from the irregular powders had higher density, bending strength, and modulus than those obtained from spherical powders [40].

The experience of the research team shows that often the properties of powders, e.g., particle size, specific surface area, or even composition, in the delivery state significantly differ from the properties depending on the production batch or supplier in relation to their parameters described in the material data sheets. Therefore, the article presents the results of a comparison of five grades of Al_2_O_3_ ceramic powder, taking into account sinterability and final product properties, in order to assess the possibility of their subsequent use in classic or additive manufacturing technology. Additionally, 3D printing tests of one of the tested powders were carried out, which was characterized by the most optimal parameters for subsequent use in the serial production of ceramic elements.

The research conducted in this work was aimed at improving the procedure for selecting a material for 3D printing using powder bed fusion technology. The detailed characterization of both chemical, physical, and technological properties of powders is important in terms of their potential applications. The correct selection of powder for incremental manufacturing technology is important from an economic point of view. The 3D printing process itself is energy-intensive and time-consuming. Therefore, choosing the optimal powder material for additive manufacturing not only saves costs and 3D printing time but also increases the chance of obtaining high-quality parts.

## 2. Materials and Methods

Five types of Al_2_O_3_ powders supplied by the manufacturer ALMATIS GmbH (Ludwigshafen, Germany) and marked as A16SG, CT3000SG, CT1200SG, CT530SG, and CL370 were used in the research. The chemical composition of the powders is shown in Table 1. Alumina powders have a high purity of more than 99.5%. They contain impurities such as Na_2_O, MgO, SiO_2_, CaO, and Fe_2_O_3_, in amounts not exceeding 0.1% each. The impurity with the highest proportion is Na_2_O, which is the result of a powder manufacturing technique in which NaOH is added to Al_2_O_3_. The presence of sodium in alumina can adversely affect the density and microstructure of the material. MgO, on the other hand, is often introduced either as an inhibitor of abnormal grain growth or as a sintering additive. The addition of Mg^2+^ and Fe^3+^ promotes a denser structure. Both Na^+^ and Mg^2+^ significantly increase viscosity. In general, impurities affect slip viscosity [45]. Keeping the viscosity of ceramic powders as low as possible is important for obtaining printed parts of high quality and accuracy [46].

Diffraction tests were carried out on a PANalytical Empyrean X-ray diffractometer (Malvern, UK) with a copper anode lamp (λCu Kα1 = 1.5419 Ǻ), a nickel filter, and a PIXcel3D counter. Measurements were carried out in the angular range of 10–110° with a measuring step of 0.013° and an exposure time of 0.5 s/step. The phase analysis of the powders was carried out on the basis of measurements made in the Bragg–Brentano geometry, using the PANalytical High Score Plus software (version: 4.8, Malvern Panalytical B.V., Almelo, The Netherlands) integrated with the ICDD PDF4 + 2020 crystallographic database.

The analysis of particle size distribution was carried out using the laser diffraction method on the BECKMAN COULTER LS ™ 13 320 MW device (Brea, CA, USA) at ITS Science Sp. z o.o. sp. k. In order to obtain optimal dispersion, the Al_2_O_3_ powder was investigated in wet mode using 20 mL of 2% sodium polyphosphate solution. The measurement time of the sample was 90 s including polarized intensity differential scattering (PIDS) technology, and the obscuration was set at 4%. Each grade of powder was tested in three repetitions.

The specific surface area was determined as a function of relative pressure with the BET (Brunauer–Emmett–Teller) and BJH (Barrett–Joyner–Halenda) methods, using a physical sorption analyzer, Quantachrome Autosorb iQ-MP (Anton Paar Company, Graz, Austria). The sample degassing process was carried out at three temperatures: 80 °C for 30 min, 120 °C for 30 min, and 350 °C for 300 min. Measuring cells with an outer diameter of ø 6 mm (wall thickness 1 mm) were used, without a filler rod. Volume measurements of nitrogen adsorption and desorption were carried out at relative pressures (p/p0) in the range from 1∙10^−6^ ÷ 0.995 for 67 measuring points. The pore volume and the average pore size were determined by nitrogen adsorption/desorption using the BJH (Barrett–Joyner–Halenda) technique [47]. The results were analyzed using the ASiQwin software (version 5.21).

Particle morphology observations were performed using the JSM 6460LV scanning electron microscope (SEM) from Jeol (Tokyo, Japan). The samples were placed on conductive carbon adhesive tape and covered with gold.

The sinterability of the powders was analyzed with the spark plasma sintering (SPS) system, type FCT HP D5 produced by FCT Systeme GmbH (Frankenblick, Germany). The powders were sintered in graphite matrices at a temperature of 1500 °C for 10 min under an argon atmosphere. During sintering, a pressure of 35 MPa was used between the punches. The heating and cooling rate was 100 °C/min. During each process, the value of the pressing punch shift was recorded, and, on this basis, the sinterability curves of the materials were then determined.

The samples after sintering using the SPS method were subjected to the apparent density test in accordance with [48]. Young’s modulus of materials was determined using the ultrasonic method on the basis of the measured values of the velocity of propagation of the longitudinal and shear waves and the density of materials according to dependence:

$$E=\rho C_{T}^{2}\frac{3C_{L}^{2}-4C_{T}^{2}}{C_{L}^{2}-C_{T}^{2}}$$

Symbol explanation:

*E*—Young’s modulus,

*ρ*—density,

*C_L_*—velocity of longitudinal wave propagation,

*C_T_*—velocity of shear wave propagation.

The analysis was carried out with the use of the Panametrix EPOCH-3 ultrasonic flaw detector (Waltham, MA, USA), and the calculations were performed with the Modulus 1.0 software.

A laboratory shaker Ø300 mm LPzE-3e (MULTISERW-Morek, Brzeźnica, Poland) with meshes of 1000, 600, 500, 250, and 100 µm was used to sieve the agglomerated powders. Then, 3D printing tests using the Innovent+^®^ (ExOne, North Huntingdon Township, PA, USA) 3D printer, were carried out on individual fractions powders, which showed the ability of printing without prior preparation. The binding material was Aqueous Binder (BA005) from ExOne, containing ethynediol and 2-butoxyethanol in amounts ranging from 2–20%. The following printing parameters were used: saturation 85%, recoat speed 150 mm/s, roller speed 300 rpm, and layer thicknesses were twice the mesh size of the sieve. Cube-shaped samples with a side of 10 mm were produced. The powdered material was spread on the working area layer by layer and selectively bonded using a binder. Each layer was heated to about 50 °C to bind the powder. After the printing process, the samples were annealed in a laboratory dryer (BINDER FD 56, Germany) at 210 °C for 4 h to cure the binder. The final process is sintering in a high-temperature oven to obtain maximum strength properties.

All measurements were performed with at least three repetitions. The repeatability of the result of measurement was below 5%.

## 3. Results and Discussion

**Figure 1:**
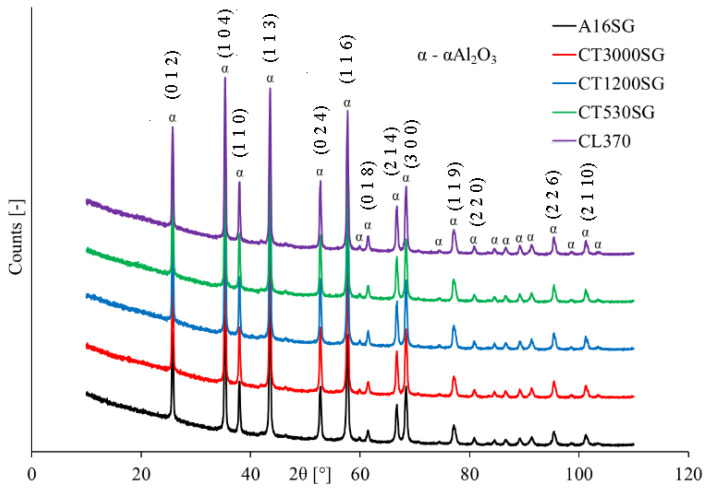
XRD patterns of five investigated grades of Al_2_O_3_ powders.

Figure 1 shows X-ray diffraction (XRD) patterns of five investigated grades of ceramic powders.

Regardless of the grades of Al_2_O_3_ powder, the recorded XRD patterns showed the highest peaks at 2θ of 26°, 35°, 38°, 43°, 57°, 61°, 66°, 68°, 77°, and 95°, which are indexed as α-Al_2_O_3_. The formula confirms that the detected reflections concerned the Miller indices (012), (104), (110), (113), (116), (018), (214), (300), (119), and (226), respectively (Card No. 04-015-8995 from the ICDD database). Chen et al. [49] and Álvarez et al. [36] in their works adjusted the α-Al_2_O_3_ phase to the diffraction effects appearing at similar angular positions.

The measurement results of the particle size distribution by the laser diffraction method, as well as specific surface area, pore volume, and average pore diameter determined on the basis of physical sorption measurements, depending on the Al_2_O_3_ powder grade were presented in Table 2.

It is well known that laser diffraction results are usually reported on a volume basis, so the volume mean D_(4,3)_ can be used to define the central point of the particle distribution. However, the median value (D_50_) defined as the value where half of the population is located above, and half below this point is more frequently used using this technique. When the result in the software will be converted to a surface area distribution, the mean value displayed is the surface mean, D_(3,2)_. The mode is the peak of the frequency distribution, the highest peak on the distribution curve. Moreover, for symmetric distributions, all central values, mean, median, and mode, will be equivalent. The symbol D_90_ means that 90 percent of the distribution lies below this value, and similarly, D_10_ means that 10 percent of the population lies below this value. On the basis of the obtained results, it was found that the CT3000SG grade had the smallest D_50_ particle size—0.560 µm. An almost three times larger powder particle size was measured for A16SG and CT1200SG, D_50_ was 1.340 µm and 1.652 µm, respectively. The biggest D_50_ particle size had CL370—3.448 µm. The studies also showed that 90% of the size of CT3000SG and CT1200SG particles do not exceed 5 µm, while the CT530SG and CL370 grades are not larger than 10 µm. The A16SG powder was characterized by the largest particles D_90_—19.640 µm. Moreover, the CT1200SG powder—span 1.507 showed the narrowest distribution width, and it increased successively for the CL370, CT530SG, and CT3000SG powders. Furthermore, the width of the distribution of the A16SG powder was almost ten times that of the CT1200SG and almost twice that of the CT3000SG.

It is well known that the driving force of the sintering process is the surface energy of the powder, which is closely related to the powder particle size. Generally, the finer particles have a higher sinterability compared to the coarser particles. Thus, by using a coarser powder, it is possible to reduce sintering shrinkage. However, the use of powders with mean particle sizes above 50 μm can cause recoating problems in additive manufacturing techniques [50,51]. Moghadasi et al. [52] confirmed in their work that larger particles improve the powder flowability, which causes a slight increase in the density of the samples. At the same time, larger powders reduce their sinterability, which ultimately leads to a decrease in density. On the other hand, the use of finer powder particles increases the mechanical strength of the printed and sintered samples. Moreover, it was found that the A16SG and CT3000SG powders had the highest specific surface area, regardless of the method used for their determination, amounting to 9.298 m^2^/g and 8.477 m^2^/g, respectively. On the other hand, CL370 and CT1200SG powders had almost three times the lower specific surface area. Smith et al. [50] for alumina powders measured a slightly higher specific surface area value of 12.05 m^2^/g. While the green sample compressed from the powder gave a value between 8.8 m^2^/g to 12.0 m^2^/g. The difference between these values is attributed to the particle-particle interfaces. It is expressed as the ratio of the particle-particle contact area to the total particle area [50]. In the work of Bechteler et al. [51], the CT3000SG and CT1200SG powders appeared. The information on the BET specific surface area for these materials was consistent with the results obtained for the analyzed five grades of Al_2_O_3_ powders. Moreover, powders with a high specific surface area, A16SG, and CT3000SG, were characterized by the largest pore volume share, almost three times higher than the other tested Al_2_O_3_ powder grades. However, this relationship did not have a significant impact on the mean pore diameter, which, regardless of the grade of the analyzed powder, ranged from 7.2 nm to 8.3 nm.

**Figure 2:**
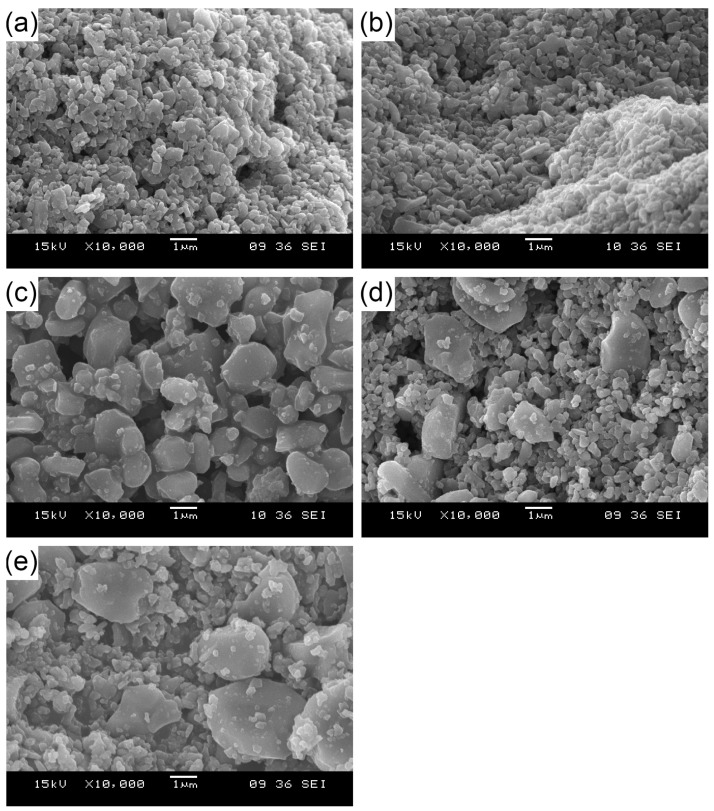
SEM images of Al_2_O_3_ grade powder particles: (**a**) A16SG, (**b**) CT3000SG, (**c**) CT1200SG, (**d**) CT530SG, (**e**) CL370.

Figure 2 shows representative examples of the particle morphology of the investigated five grades of Al_2_O_3_ powders.

The SEM observation confirmed that the variability of particle size of Al_2_O_3_ powders depends on the grade. Moreover, it has been disclosed that the powders, as supplied, tend to form agglomerates.

It is well known that the powder morphology determines the flowability and packing density of the powder. Irregularly shaped powders are beneficial for powder pressing, which also has an impact on the kinetics of powder densification during sintering [41,52]. Kim et al. [53] reported that the sintered parts, which consisted of spherical particles, were characterized by a microstructure independent of the observed direction. In contrast, the sintered parts made of the flake-shaped powders had a different microstructure in the pressing direction and another in the direction perpendicular to the pressing direction. Moreover, in the case of fine powder particles, adhesive forces play an important role. Depending on the synthesis temperature, the particles in the soft agglomerates sinter and form harder aggregates. Agglomerates are weakly bound particles that stick together under the van der Waals forces. A certain amount of aggregation can be useful to increase the flowability of the fine powder. However, if the agglomerates do not disintegrate during densification, their size can determine the sintering kinetics and cause microstructural heterogeneity [54].

**Figure 3:**
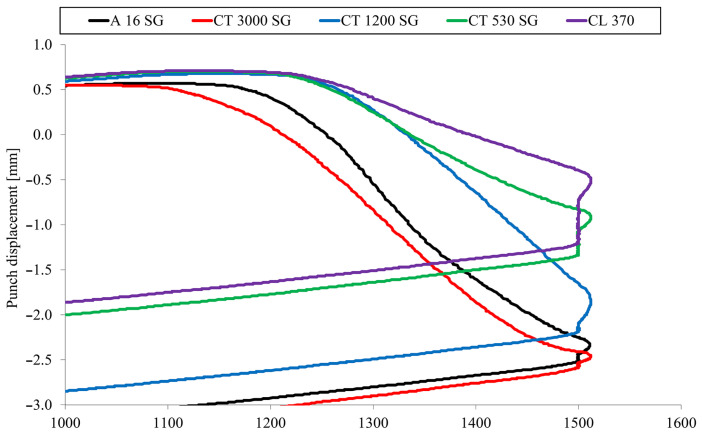
Displacement of the punch during the SPS process depending on the temperature for Al_2_O_3_ grade powder.

The registration of the position of the pressing punch during the SPS process allowed for the determination of sinterability curves for each of the investigated grades of Al_2_O_3_ powder (Figure 3). Generally, all grades of the analyzed powders showed good sinterability at 1500 °C. On the basis of the obtained results, it was found that the highest sintering shrinkage was recorded for the CT3000SG and A16SG powders, respectively. On the other hand, CL370 and CT530SG powders showed more than 50% less shrinkage.

**Figure 4:**
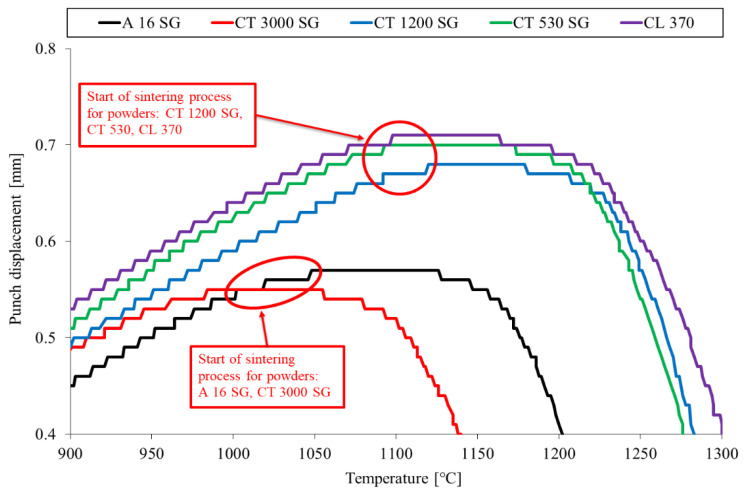
Zoom of the selected part of Figure 3 showing the initial stage of the sintering process of investigated grades of Al_2_O_3_ powders.

A detailed analysis of the displacement curves as a function of heating allowed to determine the initial stage of the sintering process for all investigated grades of Al_2_O_3_ powders (Figure 4).

The CT3000SG and A16SG grades from all of the investigated powders were characterized by the lowest sintering temperature, respectively, 980 °C and 1050 °C. On the other hand, the remaining analyzed grades of Al_2_O_3_ powder showed higher and very similar sintering start temperature values, amounting to 1100–1120 °C.

It is generally known that the sintering process takes place by atomic diffusion via grain boundaries, surfaces, and lattice. Surface diffusion is considered a non-densifying mechanism. However, it may contribute to densification in the initial stage of sintering. In the intermediate and final stages of the sintering process, densification takes place by diffusion at the grain boundary and lattice diffusion. The diffusion at the grain boundary predominates at relatively low temperatures. Therefore, such a mechanism will likely take place during the sintering of Al_2_O_3_ below 1500 °C, taking into account its high melting point (T_m_ = 2072 °C) and the slow lattice diffusivity of Al and O [55,56]. The sintering temperature determined on the basis of the performed tests is consistent with the results obtained by other researchers. For example, Yang et al. [56] investigated sintering with a constant heating rate and two-step sintering of ultrafine α—Al_2_O_3_ nanopowders at different heating rates. The researchers obtained a high sintering rate, high sintering activity, and a low sintering temperature of around 1000 °C.

The results of the apparent density and Young’s modulus of sintered Al_2_O_3_ by the SPS method for each of the investigated powder grades were presented in Table 3.

On the basis of the obtained results, it was found that, regardless of the grade of Al_2_O_3_ powder, the sinters were characterized by a density similar to the theoretical one, amounting to 99.1–99.3%. Moreover, the measured value of Young’s modulus did not show significant differences between the variants of the analyzed sinters and was consistent with the guidelines specified in [57].

**Figure 5:**
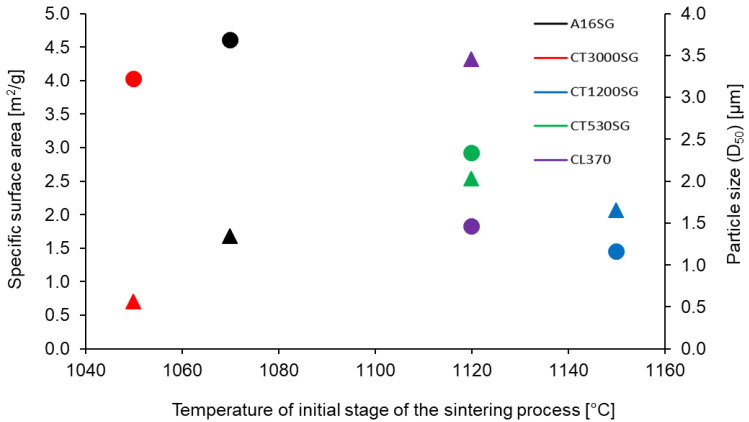
Influence of the specific surface area and mean particle size on the initial stage of the sintering temperature depending on the Al_2_O_3_ powder grade used. Circle—specific surface area, triangle—particle size (D_50_).

In order to assess the influence of the powder particle size and specific surface area on the beginning of the sintering temperature, depending on the type of the used Al_2_O_3_ powder grade, the values determined during the tests were presented in Figure 5.

On the basis of the obtained results, it was confirmed that alumina powder grade characterized by smaller particle sizes with a simultaneous greater specific surface area tends to initiate sintering processes at a lower temperature. On the other hand, the width of the particle size distribution affects the kinetics of the sintering process. Stolyarov et al. [9] showed that the wider the particle size distribution, the more intensively the compaction of the material in the initial sintering phase occurs, but at the end of the process, it slows down significantly.

It is well known that the process of printing in binder jetting technology is carried out in a layer-by-layer manner in the working area. The first step to start the printing process is to evenly apply a thin layer of powder from the ultrasonic hopper. The powder passes through a sieve of a certain size and falls on the working field of the printer. Then it is evenly distributed with a roller. However, due to the tendency of powders to absorb moisture from the environment and thus their agglomeration, and their different shape and size, not all powders meet the criterion for even layer formation and thus their use in this technology. Therefore, the first printing attempts from powders as supplied, regardless of the grade of the tested powder, failed. Despite the use of maximum ultrasonic parameters as well as the use of a sieve with the largest mesh, it was not possible to cover the working area with an even layer of powder. Therefore, for further tests, the powders were first sieved into different size fractions (Table 4) and then annealed.

**Figure 6:**
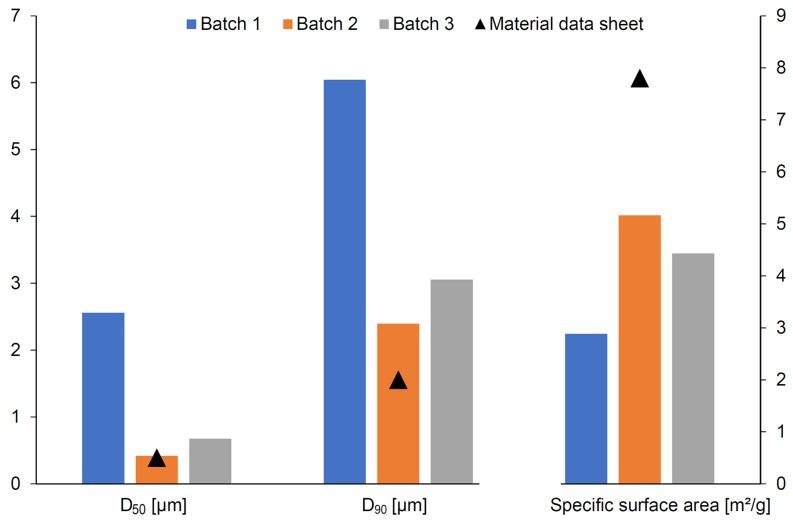
Comparison of D_50_, D_90_, and the specific surface area from three different production batches with the catalog data sheet of CT3000SG powder.

The results presented in Table 4 show that for each of the agglomerated alumina powder grades, the largest fraction was 250–500 µm, followed by 100–250 µm. For A16SG, CT3000SG, CT530SG, and CL 370 powders, more than 70% of the particles were smaller than 500 µm. Using the particle fraction in the range of 250–500 µm, for each of the tested grades of aluminum powder, the attempts to evenly distribute its layer on the print bed area were repeated. It was only possible for powder CT3000SG. Therefore, only this powder grade was selected for further research. However, it was observed that the printing parameters had to be modified depending on the delivery batches of the powder. A comparison of particle size (D_50_, D_90_) and specific surface area from three different production batches with the catalog data sheet showed significant divergence (Figure 6). It was found that between deliveries, the particle size was twice or even four times larger than it should be, and the specific surface area was almost twice as small as expected.

**Figure 7:**
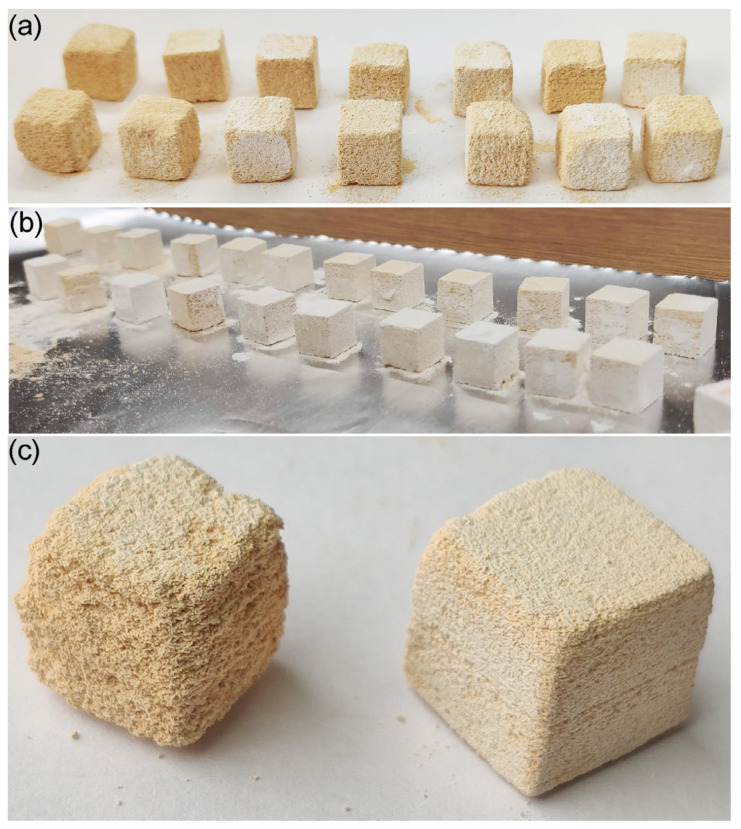
Samples printed from CT3000SG powder agglomerates by binder jetting technology from the fraction range (**a**) 500–250 µm, (**b**) 250–100 µm, (**c**) view of a representative sample made of powder with a larger fraction on the left side, and a smaller fraction on the right side.

As shown in Table 4, the most significant volume fractions of the powder were obtained from the range 500–250 µm and 250–100 µm. In order to analyze the influence of the applied particle agglomerate size on the quality of the printout, the printing process from both fractions was carried out. The view of the printed samples was presented in Figure 7.

It has been observed that the size of the particles used has a significant impact on the print quality. Samples made of larger particles were characterized by an inhomogeneous and rougher surface compared to prints made of a finer powder fraction. Moreover, it was found that the increase in the dimensional precision of the printout and the quality of the geometry of the shape of the samples compared to the designed computer model was obtained after using smaller particles of Al_2_O_3_ powder. Furthermore, with the increase in the size of the Al_2_O_3_ powder particles used, not only the roughness of the sample, but also its porosity should increase. Therefore, if the key feature will be the need to control the porosity of the product, and the edges of the sample will not play a crucial aspect or can be modified in post-processing operations, this method should allow control of this parameter. This can be particularly useful for some applications, such as in the chemical industry or medicine.

## 4. Conclusions

The presented results clearly indicate that both the particle size and the specific surface area had a significant impact on the sintering process of the Al_2_O_3_ powder grades. The lowest sintering temperature was characteristic for CT3000SG and A16SG powders, while the highest was for CT1200SG powder. Generally, the greater the surface development and the smaller particle size of the Al_2_O_3_ powder and the wider their distribution, the lower the temperature of the initial stage of the sintering process. These results could be crucial for the correct design of the classical sintering process for Al_2_O_3_ powders materials produced by die-forming as well as by additive processes. Differences between the catalog data sheet and the results of the measurements were also determined. Due to the specificity of the printing process, it was shown that not all of the analyzed Al_2_O_3_ grades of powder were suitable for use in this technology. Moreover, it was necessary to properly prepare the powder, by sieving and annealing, before the printing process. On the basis of the obtained results, it was found that by selecting the appropriate powder fraction, it was possible to control the quality of the printout, defined as the accuracy of shape geometry, surface roughness, and porosity. Controlling these characteristics can be crucial for various industrial applications. In the future, it is planned to focus research on the analysis of parameters and the method of sintering printed samples, depending on the powder fraction used. This should allow for controlling the final properties of Al_2_O_3_ products produced by the additive binder jetting method.

Summarizing the results obtained for the Al_2_O_3_ powders analyzed, the best experimental results were obtained for the CT3000SG grade. Among all powders, CT3000SG had the smallest particle size of D50—0.560 µm, and consequently this powder has a relatively high specific surface area of 8.477 m^2^/g. In addition, the CT3000SG grade had the lowest initial sintering temperature. This powder was the only one among the five variants that distributed well on the printer’s working area through its small particle size. This indicates that it could become a potential material for additive manufacturing of ceramic parts using Binder Jetting technology.

## Figures and Tables

**Table 1:** Chemical composition of Al_2_O_3_ powders.

		Powder Grade				
		A16SG	CT3000SG	CT1200SG	CT530SG	CL370
Chemical composition [%]	Na_2_O	0.07	0.08	0.06	0.09	0.10
	MgO	0.05	0.07	0.07	0.04	-
	SiO_2_	0.03	0.03	0.05	0.03	0.03
	CaO	0.02	0.02	0.04	0.03	0.03
	Fe_2_O_3_	0.02	0.02	0.02	0.02	0.03
	Al_2_O_3_	balance	balance	balance	balance	balance

**Table 2:** Particle size distribution, specific surface area, and porosity of investigated Al_2_O_3_ powder grades.

		Powder Grade				
		A16SG	CT3000SG	CT1200SG	CT530SG	CL370
The particles size distribution	D_(3,2)_ [µm]	0.634	0.459	1.550	0.707	1.086
	D_(4,3)_ [µm]	5.840	1.865	2.049	3.351	4.518
	D_10_ [µm]	0.236	0.216	0.945	0.241	0.490
	D_50_ [µm]	1.340	0.560	1.652	2.023	3.448
	D_90_ [µm]	19.640	4.816	3.435	7.515	9.552
	Moda [µm]	0.326	0.326	1.451	2.787	4.444
	Span (D_90_–D_10_)/D_50_	14.481	8.214	1.507	3.596	2.628
Specific surface area [m^2^/g]—BET		9.298	8.477	3.403	4.619	2.913
Specific surface area [m^2^/g]—BJH		9.233	8.698	3.573	4.983	3.057
Pore volume [cm^3^/g]		0.019	0.017	0.006	0.008	0.005
Pore average diameter [nm]		8.338	8.180	7.435	7.258	7.217

**Table 3:** Density and Young’s modulus of sintered Al_2_O_3_ depending on the grade of powder used.

	Powder Grade				
	A16SG	CT3000SG	CT1200SG	CT530SG	CL370
Density [g/cm^3^]	3.95	3.96	3.96	3.96	3.95
Young modulus [GPa]	380	385	389	390	386

**Table 4:** Al_2_O_3_ fractions after sieving depending on the grade of powder.

		Powder Grade				
		A16SG	CT3000SG	CT1200SG	CT530SG	CL370
Percentage depending on the screen size used	>1000 [µm]	0.92	0.75	0.65	1.35	1.85
	1000–600 [µm]	13.20	10.20	12.55	10.02	11.10
	600–500 [µm]	5.30	14.55	18.95	6.45	6.60
	500–250 [µm]	53.45	41.35	37.80	50.35	52.15
	250–100 [µm]	22.50	28.95	22.75	25.00	23.50
	<100 [µm]	4.63	4.20	7.30	6.55	4.80

## Data Availability

Not applicable.
